# Adhesion between Asphalt and Recycled Concrete Aggregate and Its Impact on the Properties of Asphalt Mixture

**DOI:** 10.3390/ma11122528

**Published:** 2018-12-12

**Authors:** Yueqin Hou, Xiaoping Ji, Jia Li, Xianghang Li

**Affiliations:** 1 School of Human Settlements and Civil Engineering, Xi’an Jiaotong University, Xi’an 710049, China; houyueqin527@xjtu.edu.cn; 2 Key Laboratory for Special Area Highway Engineering of Ministry of Education, Chang’an University, Xi’an 710064, China; chdli1996@163.com (J.L.); hanglx.gd@foxmail.com (X.L.)

**Keywords:** hot mix asphalt containing recycled concrete aggregate, contact angle, adhesion energy, water stability, fatigue performance

## Abstract

To study and evaluate the adhesion between recycled concrete aggregate and asphalt, the contact angles (CAs) between droplet (water and ethanol) and recycled concrete aggregate (RCA), natural aggregates, and solid bitumen (matrix asphalt, SBS modified asphalt) were tested via the sessile drop method with an optical microscope. The surface free energy was then calculated. The CAs between hot asphalt and RCA and natural aggregates were tested via the hanging slice method. The adhesive energy between asphalt and RCA and natural aggregates were calculated based on the test results of the surface free energy and CAs. Then, the influence of RCA on the water stability and fatigue performance of the asphalt mixture was analyzed by testing the water stability and fatigue properties of hot mix asphalts containing RCA (HMA-RCA) with different aggregates and RCA dosages. The surface energy of the various aggregates and the CAs between aggregates and asphalts were sorted as follows: Granite > RCA > serpentinite > limestone. The surface energy and CA of RCA were very close to that of serpentinite. The adhesive energy between various aggregates and asphalt were sorted as follows: Limestone > serpentinite > RCA > granite. The adhesive energy between RCA and asphalt was also very close to that of serpentinite. The residual Marshall stability, tensile strength ratio, and fatigue performance of the HMA-RCAs were gradually reduced along with the increasing RCA dosage. This effect may be attributed to the fact that the adhesive energy between the RCA and the asphalt was less than that of water and that the asphalt was easily stripped from the RCA surface. Excessive RCA content in the aggregate can lead to excessive porosity of the HMA-RCA. The CAs and adhesive energy between RCA and asphalt showed significant effects on the water stability and fatigue performance of HMA-RCA.

## 1. Introduction

### 1.1. Research Background

Given the constant development of human production activities, the contradiction between the supply and demand of high-quality gravels that are used in pavements becomes increasingly prominent, and the price of such products continues to increase. In addition, the construction and demolition of buildings, as well as the construction and renovation of cement concrete or asphalt concrete pavement, produce up to 600 million tons of solid wastes each year, thereby leading to a series of environmental and social problems. Therefore, recycling solid waste not only alleviates the shortage of natural aggregates, but also solves the problem of processing construction waste [1]. Recycled concrete aggregates (RCA) are less than 40 mm in particle size and are produced from waste concrete after sorting, carving, crushing, and grading. RCA can be widely used in non-load-bearing structural concrete, such as foundation cushions, gutters, drainage troughs, coastal protection dikes, and so on. The construction of asphalt concrete pavement requires a large amount of aggregates, and because high-quality aggregates are non-renewable resources, researchers hope to replace some of the natural aggregates with recycled aggregates, so RCA application in asphalt mixture has attracted the attention of researchers. At present, in asphalt pavement, RCA can be used to build the asphalt concrete surface layer, cushion layer, base layer, and so on [2,3,4].

The properties of asphalt mixture are closely related to the performance and adhesion between aggregate and asphalt. Loss of adhesion is caused by the breaking of adhesive bonds between the aggregate surface and the asphalt binder primarily because of the coupled action of water and load. Therefore, loss of adhesion leads to several distresses in the asphalt pavement, such as water damage and fatigue damage. Previous studies [5,6] have shown that RCA is composed of natural sandstone material and cement mortar with a special surface microscopic structure and chemical composition. The adhesion between RCA and asphalt is closely related not only to the chemical properties of materials, but also to their surface structure. RCA is used in cement hydration products, including C–S–H (*x*CaO·SiO_2_·*y*H_2_O), ettringite (3CaO·Al_2_O_3_·CaSO_4_·32H_2_O), single-sulfur type of calcium sulfoaluminate (3CaO·Al_2_O_3_·CaSO_4_·12H_2_O), and Ca(OH)_2_. These components have a certain activity that can lead to feeble chemical reactions with acid asphalt. The adhesion between RCA and asphalt is affected by chemical reactions. Moreover, the surface of RCA is rougher than that of natural gravels because of many micro cracks and voids. Therefore, the adhesion between RCA and asphalt demonstrates a unique behavior that complicates the relationship between the road performance of hot mix asphalt containing RCA (HMA-RCA) and the dosage of RCA. Moreover, the road performance of HMA-RCA cannot satisfy the required technical specifications. Therefore, revealing the micro-mechanism and influencing factors of the adhesion between RCA and asphalt serves as a theoretical basis for optimizing the design of HMA-RCA. 

### 1.2. Literature Review 

The recycling of recycled aggregates in asphalt mixtures has attracted research attention in recent years. Existing studies mainly focus on the road performance of HMA-RCAs, including their Marshall and volume indices, water stability, permanent deformation, and fatigue performance. At the same time, some studies have evaluated the performance of HMA-RCAs from a microscopic perspective [7,8]. Numerous studies show that RCA contains a certain amount of cement mortar with low density, large pores, rough surface, and many micro cracks.

Many studies have shown that the asphalt content of HMA-RCAs is higher than that of natural gravels due to a large amount of asphalt absorbed by the pores and micro cracks of RCAs, and it increases along with the increasing of RCA dosage [9,10,11,12]. The asphalt in the mixture can be divided into effective asphalt and asphalt absorbed by the aggregate. Although HMA-RCA contains a large amount of asphalt, its effective asphalt content is lower than that of HMA with natural gravels [12,13,14]. RCA can be divided into recycled coarse aggregate (≥4.75 mm) and recycled fine aggregate (<4.75 mm). Some studies suggest that the dosage of recycled fine aggregate must be limited to decrease the asphalt content [15] because recycled fine aggregate contains a large amount of cement mortar [16] with a large specific surface area [17] and high oil adsorption [18].

Water stability is caused by the stripping of asphalt from the aggregate surface because of the coupled actions of water and load. The water stability of HMA-RCA varies with RCA content, and the law behind such variation is very complicated. Some studies show that RCA can improve the water stability of HMA-RCA and that the water stability of HMA-RCA increases along with increasing RCA dosage [12,13,19,20,21,22]. However, other studies find that the water stability of HMA-RCA decreases with increasing RCA content [23,24]. Some studies find that the water stability of HMA-RCA does not meet the specification requirements [13], but others show opposite results [19,21,22,25]. 

In terms of resistance to permanent deformation and modulus, some studies suggest that HMA-RCA is more capable of resisting permanent deformation, meeting specification requirements [10,12,13,21,26,27,28], and demonstrating high rebound, dynamic, and bending moduli compared with traditional mixtures [13,24,26,29]. However, other studies claim that the stiffness modulus and resistance of HMA-RCA to permanent deformation decrease [30] along with increasing RCA content, and that HMA-RCA does not meet specification requirements [13,15] because its asphalt content is greater than that of traditional HMA.

The fatigue properties of HMA-RCA are identical to those of traditional HMA [12,20,25], and recycled fine aggregate can improve fatigue performance [18]. Numerous studies show that the low temperature cracking performance of HMA-RCA is reduced along with increasing RCA content [23,24,30] because the effective asphalt content of HMA-RCA decreases with increasing RCA dosage.

Few studies have investigated the micro structure of RCA and the adhesion properties between RCA and asphalt. Hou et al. [9] studied the microscopic properties of RCA by means of SEM. The crushed value, water absorption, density, and adhesion with asphalt of RCA were tested. However, the adhesion between the aggregate and the asphalt and the macroscopic asphalt mixture water stability and fatigue performance are not closely related. 

In other words, the adhesive mechanism and its influencing factors must be determined to improve the road performance of HMA-RCA. In this study, the contact angles (CAs) among droplets (water and ethanol), RCA, several natural aggregates, and solid bitumen (matrix asphalt, styrene-butadiene-styrene (SBS)-modified asphalt) were tested using the sessile drop method. The surface free energy was then calculated. The CAs among hot asphalt, RCA, and natural aggregates were tested using the hanging slice method. The adhesive energy among the asphalt, RCA, and natural aggregates were calculated based on the test results of the surface free energy and CAs. HMA-RCAs with RCA dosages of 0%, 30%, 60%, and 100% were designed, and the water stability and fatigue performance of these HMA-RCAs were tested. The effects of the adhesive on the water stability and fatigue performance were then analyzed.

## 2. Raw Materials and Mixtures

### 2.1. Raw Materials 

The 70# matrix asphalt and SBS modified asphalt were used in experiments, and the technique performances were tested and shown in Table 1 according to the Chinese Specification of JTJ 052-2000 (RIOH, 2000) [31].

Waste cement concrete was directly collected from a bridge demolishing spot and then crushed and sorted to obtain RCA. The performance of RCAs was tested and shown in Table 2 according to the Chinese Specification of JTG E42-2005 (RIOH, 2005) [32]. Three different natural gravels were utilized in the experiments for the comparative analysis, namely limestone, granite, and serpentine.

Water and ethyl alcohol were used in the CAs tests with aggregates and solid asphalts, and the parameters are shown in Table 3.

### 2.2. Mixtures

Four HMAs were designed to investigate the water stability of the mixture. The aggregate used in the asphalt mixture was RCA and limestone, and the content of RCA in the aggregate was 0%, 30%, 60%, and 100%, respectively, which were marked as M1, M2, M3, and M4. To determine the optimal asphalt content of the above HMAs, the bulk relative density, Marshall stability and flow value, air voids, voids in mineral aggregate, and voids filled with asphalt were measured according to the Chinese Specification of JTG F40-2004 (RIOH, 2004) [33]. The results are listed in Table 4.

## 3. Experimental Methods

### 3.1. CA between Aggregate and Solid Asphalt Tested with a CA Measurement Device

The CA is an important index that indicates the wetting ability of a liquid to solid. On the junction of the three phases of gas, liquid, and solid, the tangent lines of the interface between gas and liquid and those of the interface between solid and liquid can be obtained. The CA refers to the angle between two tangent lines through the liquid internal part. The smaller the CA is, the greater the wetting ability of the liquid to solid is. The CA between the asphalt and aggregate indirectly reflects the adhesion in the asphalt mixture, which is a basic parameter for calculating the adhesive energy.

**Figure 1:**
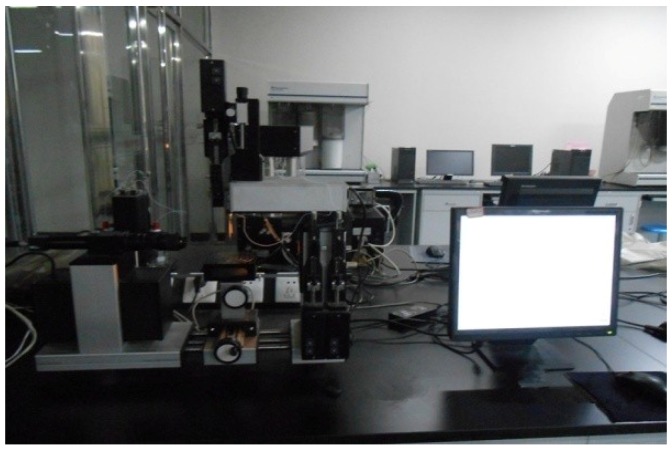
Optical CA measuring device.

This study used an optical CA measuring device that included a host computer, dosage controlling unit, an injecting unit, and a specific analysis software. The device is shown in Figure 1. Several pictures can be captured per second with a measurement accuracy of ±0.1°. Sessile drop method was selected instead of the hanging drop method or inclined plate method. In the chosen method, the droplets were dropped on the solid surface, two tangent lines were obtained, and the angle between these lines was measured. Six parallel experiments were performed for each test. The CAs among the droplets, several aggregates, and solid asphalts were surveyed using the abovementioned methods. Water and ethanol droplets were used in the experiment. The solids used in the experiment included limestone, granite, serpentine, RCA, and two solid asphalts (matrix and SBS-modified asphalts). The testing temperature was 15 °C.

**Figure 2:**
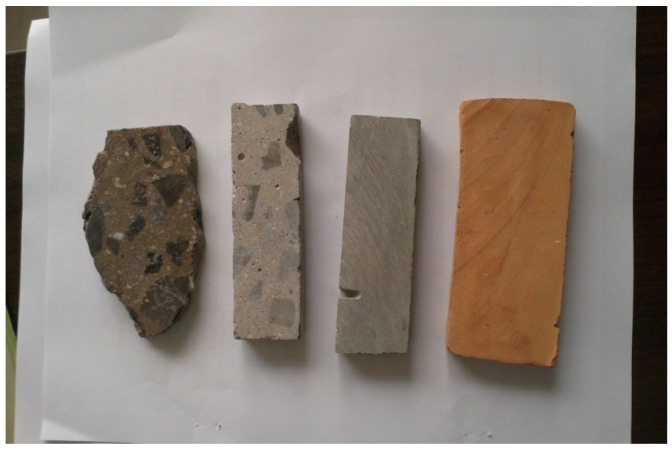
Aggregate test piece.

An aggregate is usually a particulate and lacks a flat surface, which greatly influences the measurement of surface energy parameters. To improve the accuracy, stones with the same materials as those of the aggregates were polished using a metallographic polishing machine. Different types of the aggregate test pieces are shown in Figure 2. Asphalt is a solid with a viscous state below 60 °C or at a normal temperature. Therefore, the test method for liquid materials cannot be used to survey the surface free energy of asphalt. First, a 1 mm-thick glass slide was used as a base plate, washed, and placed in an oven for drying at 60 °C for 15 min. Second, the asphalt was heated to 160 °C and sufficiently liquefied. Third, the prepared glass slide was immersed into the liquid asphalt, slowly removed, and completely adhered. The thicker portion of the asphalt was scraped off on the slide edge. Fourth, the glass slide was cooled at room temperature and stored in dry condition for more than 8 h.

### 3.2. CA between HMA and Aggregate Tested using the Hanging Slice Method

To calculate the adhesive energy between asphalt and aggregates, the CA between asphalt and aggregates must be determined. However, given the high viscosity properties, asphalt cannot be dropped to a solid surface even in a hot-melt state. Moreover, that the hot-melt asphalt will form a smooth surface on the solid surface cannot be ensured because of the favorable ductility of asphalt and the easy formation of long tar silk. Therefore, the CA cannot be easily measured using the CA measurement device. 

**Figure 3:**
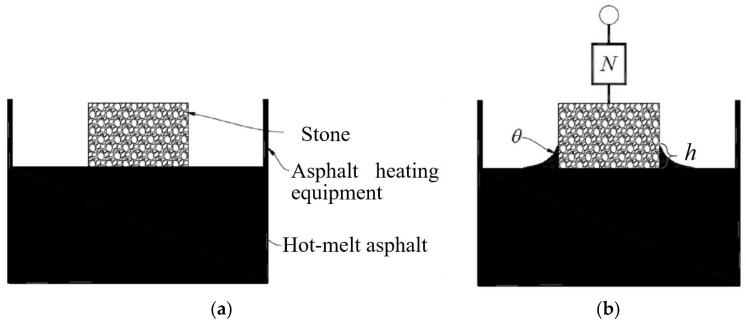
The hanging slice method: (**a**) Before the stone touches the liquid asphalt; (**b**) After the stone touches the liquid asphalt.

The hanging slice method was selected to measure the CA between hot-melt asphalt and aggregate. The testing principle is described as follows. Before the stone or aggregate touches the surface of hot-melt asphalt, the asphalt surface can be regarded as a horizontal plane. When the stone touches the asphalt surface, the hot-melt asphalt rises along with the stone to a certain distance because of the effect of surface energy and then forms a new asphalt surface, as shown in Figure 3. The testing temperature was 160 °C. The new asphalt surface tends to contract automatically, thereby leading to surface tension, which can be detected by using the dynamometer. The angle, θ, between the direction of surface tension and the stone surface denotes the CA between the hot-melt asphalt and stone. The relationship of the surface energy of hot-melt asphalt (*γ_a_*), with the CA between the hot-melt asphalt and stone (*θ*) and the force value (*N*), satisfies Equation (1) as follows:

(1) $$\gamma_{a}\cos\theta =\frac{N-mg}{2(b+c)},$$

where:

*γ_a_* is the surface energy of the hot-melt asphalt, 10^−3^ J/m^2^.
*θ* is the CA between the hot-melt asphalt and aggregate, °;
*N* is the force value, N;
*m* is the weight of the stone plate, g; and
*b* and *c* are the length and width of aggregate, respectively, cm.

### 3.3. The Calculation Method of Surface Free Energy and CA 

Young equation [34] can be used to described the relationship between the CA and surface free energy between the solid-liquid interface:

(2) $$\gamma_{s}-\gamma_{sl}=\gamma_{l}\cos\theta ,$$

where *γ_s_*, *γ_sl_*, and *γ_l_* are the surface free energies of the solid, solid-liquid interface, and liquid, respectively, N; and *θ* is the CA between the solid-liquid interface, °.

The adhesive energy in the adhesion process is equal to the variation of the Gibbs free energy per unit surface area, which can be calculated as follows. We can use the variation of the Gibbs free energy per unit surface area to describe the adhesive energy between the aggregate and the asphalt during the adhesion process. The formula is as follows:

(3) $$W_{a}=-\Delta G=\gamma_{l}-\gamma_{sl}+\gamma_{s},$$

The adhesive energy can be calculated as follows according to Equations (2) and (3):

(4) $$W_{a}=-\Delta G=\gamma_{l}(\cos\theta +1),$$

The adhesive energy between asphalt and aggregate can be thought to be composed of two parts, namely, the dispersion component and polar component when combining van der Waals force theory and Lewis theory on acid and alkali, as well as by ignoring the small force between the molecules:

(5) $$W_{a}=2\sqrt{\gamma_{s}{}^{d}\gamma_{l}{}^{d}}+2\sqrt{\gamma_{s}{}^{p}\gamma_{l}{}^{p}}),$$

where *γ_l_^d^* and *γ_l_^p^* are the dispersion and polarity components (acid-alkali effect) of the surface free energy of liquid, respectively; *γ_s_^d^* and *γ_s_^p^* are the dispersion and polarity components (acid-alkali effect) of the surface free energy of solid, respectively.

According to Equations (4) and (5), the adhesive energy can be calculated as follows:

(6) $$W_{a}=\gamma_{l}(\cos\theta +1)=2\sqrt{\gamma_{s}{}^{d}\gamma_{l}{}^{d}}+2\sqrt{\gamma_{s}{}^{p}\gamma_{l}{}^{p}}),$$

### 3.4. Water Stability Test

Residual Marshall stability (RMS) is among the indices used to evaluate the water stability of an asphalt mixture. A large RMS value indicates the favorable water stability of asphalt mixtures. For each mixture type, eight specimens measuring Φ 101.6 mm × 63.5 mm were fabricated with 75 times of two-face compaction. These specimens were divided into two groups. The stability of the first group, MS_0_, was tested after being immersed in a 60 °C water bath for 30 min to 40 min, whereas the stability of the second group, MS_1_, was tested after being immersed in a 60 °C water bath for 48 h. The immersion RMS was then calculated using Equation (7). The evaluation method is similar to that specified in ASTM D 1559 (Determining the Marshall Stability of Bituminous Mixture):

(7) $$\mathrm{RMS}=\frac{{\mathrm{MS}}_{1}}{{\mathrm{MS}}_{0}}\times 100,$$

where:

RMS is the residual Marshall stability, %;
MS_1_ is the stability after 48 h immersion, kN; and
MS_0_ is the stability after 30 min immersion, kN.

TSR is another index for evaluating the water stability of asphalt mixture. A large TSR value indicates the favorable water stability of asphalt mixtures. For each mixture type, eight specimens measuring Φ 101.6 mm × 63.5 mm were fabricated with 50 times of two-face compaction. The specimens of group 1 were saturated under a 730 mm Hg vacuum condition for 15 min, placed in sealed plastic bags containing 10 mL water, placed in a refrigerator with a constant temperature of −18 °C for 16 h, taken out of the plastic bags, and immersed in a 60 °C water bath for 24 h. The specimens of both groups 1 and 2 were then tested to obtain their tensile strength after being immersed in a 25 °C water bath for 2 h. TSR was calculated with Equation (8). Although this testing method is similar to that specified in AASHTO T 283-03 (Standard Method of Test for Resistance of Compacted Asphalt Mixtures to Moisture-Induced Damage), the experiment parameters were slightly different:

(8) $$\mathrm{TSR}=\frac{{\mathrm{ITS}}_{1}}{{\mathrm{ITS}}_{0}}\times 100,$$

where:

TSR is the tensile strength ratio, %;
ITS_0_ is the tensile strength without freeze-thaw circles, MPa; and
ITS_1_ is the tensile strength under freeze-thaw circles, MPa.

### 3.5. Fatigue Test

**Figure 4:**
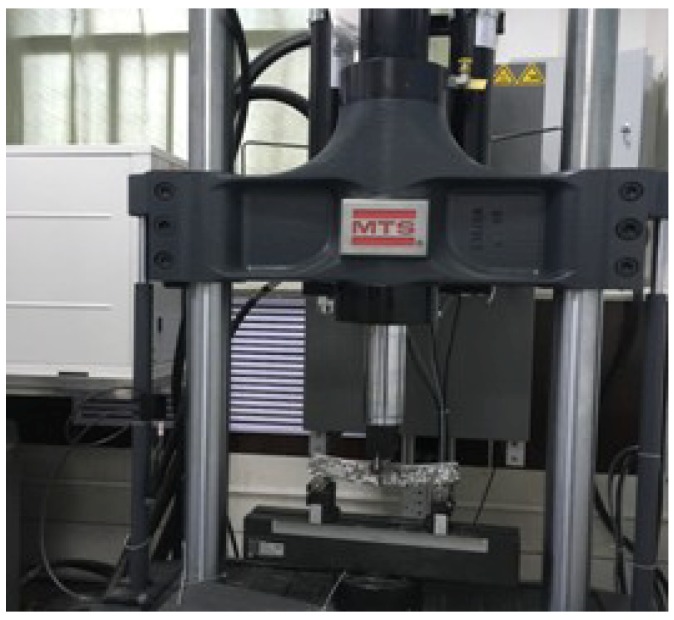
MTS Landmark (MTS 810).

**Figure 5:**
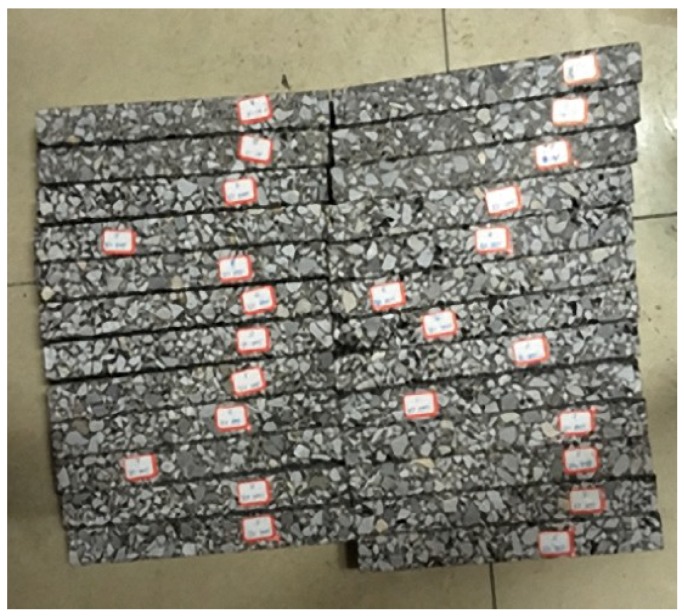
Standard trabecular test pieces.

According to the Chinese Specification of JTG E20-2011 (RIOH, 2011) [35], granite, serpentinite, limestone, and RCA were used as aggregates, 300 mm long, 300 mm wide, and 50 mm thick and were prepared by a wheel roller forming instrument. When the temperature of the test piece dropped to room temperature, it was cut into standard trabecular test pieces with a length of 250 mm, a width of 30 mm, and a thickness of 35 mm. In order to calculate the corresponding load at each stress level in the fatigue test, the trabecular bending test was carried out. The loading speed was 10 mm/min, and the maximum load of the standard trabecular test piece was obtained. The aggregate types and numbers of different asphalt mixtures are shown in Table 5. Mechanical testing and simulation (MTS) was adopted to test the fatigue performance of the HMA-RCA specimens under the stress-control mode. Three stress levels were used, which were 80%, 75%, and 70% of the maximum load, respectively. The loading frequency was 15 Hz, and the testing temperature was 15 °C. The MTS and standard trabecular test pieces are shown in Figure 4 and Figure 5.

## 4. Results and Discussion

### 4.1. CA and Surface Energy Parameters of Aggregates and Solid Asphalt

#### 4.1.1. CA

The CAs among the aggregates, solid asphalts, water, and ethanol were tested using a CA measurement device. Table 6 presents the results, from which the following can be deduced. The CAs among various aggregates and water are sorted as follows: Limestone > serpentinite > RCA> granite. The CA between the RCA with water is very close to that of serpentinite. The CA between asphalt and water is close to 90°, which indicates that water and asphalt are two different materials with poor compatibility and the water shows a stronger polarity than asphalt. Considering the opposite polarity of water and asphalt, those materials with favorable wettability to water show a poor wettability to asphalt. The experiment results indicate that water has the best wettability to granite and the worst wettability to limestone. From this, it can be inferred that limestone has the best wettability to asphalt. Serpentinite and RCA have a moderate level of wettability to asphalt.

#### 4.1.2. Surface Free Energy

According to the parameters of the surface energy of water and ethanol (Table 3) and the CAs between aggregates and asphalts (Table 6), the surface energy parameters between various solid bitumens and aggregates can be solved with Equation (8), as shown in Table 7. The results indicate the following:

(1) The polar components comprise the main part of the surface energy of various aggregates and are much greater than the dispersion components. The surface energy of aggregates is sorted as follows: Granite > RCA > serpentine > limestone.
(2) The surface energy of the modified asphalt is greater than that of the matrix asphalt.

### 4.2. CA between Liquid Asphalt and Aggregate

Table 8 present the test results of CAs between hot-melt asphalt and aggregate. The results indicate the following:

(1) A small CA indicates the good wettability of asphalt to aggregate. The CA between various aggregates and the same melt-asphalt is sorted as follows: Granite > RCA > serpentine > limestone. The surface energy and CA of RCA and serpentinite are very similar to each other.
(2) The CA between the SBS-modified asphalt and the aggregate is greater than that of the matrix asphalt because decreasing the light oil content in modified asphalt decreases its infiltration property, thereby increasing the CA of modified asphalt.

It can be seen from the CA test that the CAs between different aggregates and water or asphalt were different. This may be due to the different chemical composition of the different aggregates. Related studies have shown that the CA between asphalt and aggregate has a close relationship with the content of elements, such as Si, Al, Ca, and Mg, on the aggregate surface, and the composition of the chemical composition determines the acidity and basicity of the aggregate to a certain extent, thereby affecting the chemical reaction with the asphalt.

Limestone has a high content of Ca and Mg, a low SiO_2_ content, an alkaline aggregate, the surface is easily infiltrated by asphalt, and its CA is lower with asphalt, while granite is the opposite, the SiO_2_ is high and an acidic aggregate, so the same acidic asphalt is difficult to infiltrate. Although RCA has a complex chemical composition, the RCA surface contains a small amount of cement hydration products, including CSH, ettringite, monosulfide-type sulphoaluminate, and Ca(OH)_2_. These components have a certain activity and can cause weak chemical reactions with acidic asphalt. Therefore the CA between RCA and asphalt is between limestone and granite.

### 4.3. Adhesive Energy between Asphalt and Aggregate

According to the parameters of the surface energy of asphalt (Table 7) and the CAs between aggregates and liquid asphalts (Table 8), the adhesive energy of liquid asphalts and aggregates can be calculated with Equation (8), as shown in Table 9. The results indicate the following:

(1) A great adhesive energy indicates the excellent adhesion between aggregate and asphalt. When the same asphalt is used, the adhesive energy of different aggregates is sorted as follows: Limestone > serpentine > RCA > granite. The adhesion between RCA and asphalt is similar to that of serpentinite.
(2) The adhesive energy between modified asphalt and aggregate is greater than that of matrix asphalt because the SBS-modified asphalt has a high surface free energy.

### 4.4. Water Stability and Fatigue Performance

#### 4.4.1. Water Stability

**Figure 6:**
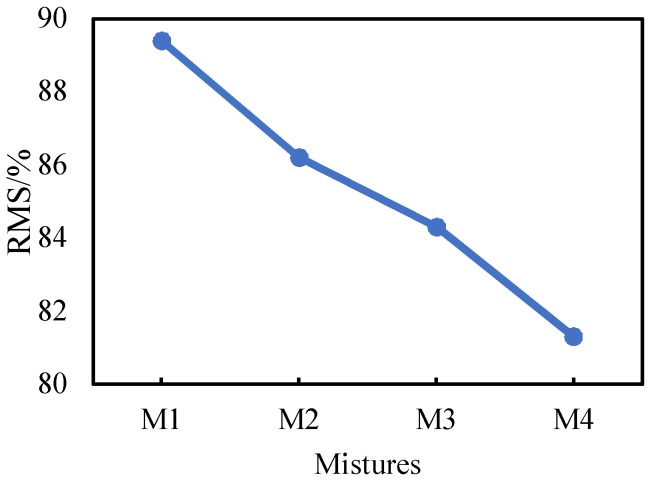
RMS Results.

The adhesive energy between RCA and asphalt is less than that of serpentine according to the results of CA and adhesive energy. Therefore, the water stability of HMA-RCAs are worse than those of the traditional mixture. Figure 6 shows the RMS results of the four HMA-RCAs. The RMS values of M1, M2, M3, and M4 are 89.4%, 86.2%, 84.3%, and 81.3%, respectively. The RMS of HMA-RCAs decreases gradually as the RCA content increases because the adhesive energy between asphalt and RCA is less than that of limestone, and the CA between RCA and asphalt is greater than that of water. So, the asphalt spalls more easily from the RCA surface under water, resulting in a decrease in water stability of the asphalt mixture.

**Figure 7:**
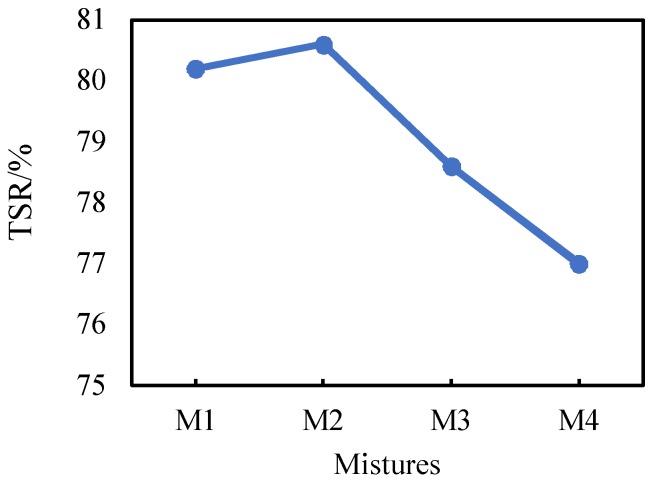
TSR Results.

Figure 7 shows the TSR results. The TSR values of M1, M2, M3, and M4 are 80.2%, 80.6%, 78.6%, and 77.0%, respectively. The TSR of HMA-RCAs gradually decreases as the RCA content increases, which varies with the same law as that of RMS. When the RCA content is increased, the water stability of the asphalt concrete is generally weakened. However, it can be found from the TSR test results that when the RCA content was increased from 0% to 30%, the water stability of asphalt concrete was slightly increased by 0.4%, which may be due to experimental errors.

#### 4.4.2. Fatigue Performance

**Figure 8:**
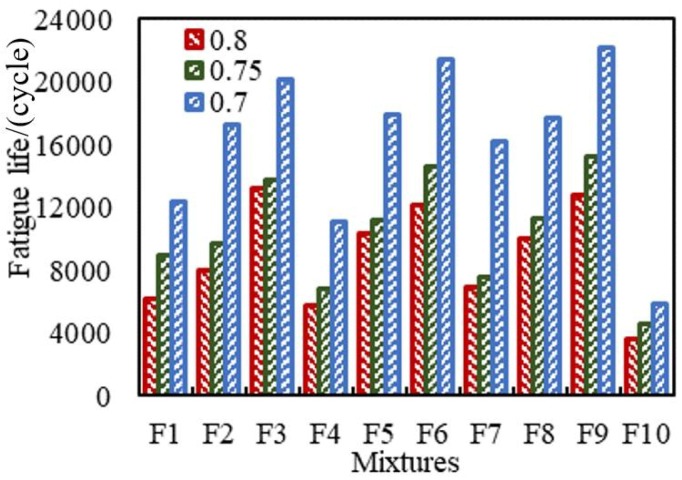
Curves of the fatigue life.

Table 10 shows the fatigue test result of HMA-RCA. It can be seen from Table 10 that as the stress level increases, the fatigue life of all standard trabecular specimens decreases rapidly. Moreover, as the RCA dosage increases, the fatigue life of the test piece also decreases significantly. When the content of RCA (F10) is 100%, the fatigue life of the test piece is only 30% of that of the ordinary aggregate test piece (F3; 6; 9). The fatigue life of standard trabecular specimens with different granite, serpentinite, limestone, and RCA contents under different stress levels is shown in Figure 8.

The adhesion of aggregate and asphalt, and the aggregate strength characteristics and the porosity of asphalt mixture are the main factors affecting the fatigue performance of asphalt mixture. It can be concluded from Figure 8 that the fatigue life of the test pieces with the aggregate as limestone (F7–9) is higher, the serpentinite (F4–6) is the second highest, and the fatigue life of the specimen with granite (F1–3) is the lowest, regardless of the load level. This phenomenon is mutually confirmed by the results of the CA test and adhesion energy calculation between different aggregates and asphalt. From the above test results, limestone has the best adhesion to asphalt, followed by serpentine and granite. This is because when the adhesion between the aggregate and the asphalt decreases, the intermolecular van der Waals force on the surface of the asphalt and stone decreases, and the asphalt mixture loosens, resulting in a decrease in the shear strength and bond strength of the asphalt mixture, ultimately leading to a decrease in fatigue performance.

It can be seen from the test results that although the CA and adhesion energy of RCA are very similar to the CA and adhesion energy of serpentine, when the RCA content in the aggregate increased, the fatigue life of the test pieces were significantly reduced, and when the RCA content was 100%, the fatigue life of the test pieces were the lowest. This is because, in addition to adhesion, the strength characteristics of the aggregate and the void ratio of the asphalt mixture also affect the fatigue performance. From the test results of the aggregate crushing values in Table 2, it can be known that the fatigue performance of the test pieces was poor due to the insufficient compressive strength of the RCA, which was easily destroyed during the fatigue performance test process. Referring to Table 4, as the RCA content increases, the porosity of the asphalt mixture also increases. The high air voids’ content causes this to reduce the fatigue life of the test piece.

## 5. Conclusions

The polar component was the major part of the surface energy of aggregates and was much greater than the dispersion component. The surface energy of aggregates was sorted as follows: Granite > RCA > serpentine > limestone.

The CA between various aggregates and the same melt-asphalt was sorted as follows: Granite > RCA > serpentine > limestone. The CA of RCA and serpentinite were very close, and activation can reduce the CA and improve wettability. When the same asphalt was used, the adhesive energy of different aggregates was sorted as follows: Limestone > serpentine > RCA > granite. The adhesion between RCA and asphalt was the same as that of serpentinite.

The RMS and TSR of HMA-RCAs decrease gradually as the RCA content increases because the adhesive energy between RCA and asphalt was less than that of water. The water stability of HMA-RCAs was closely related to the CA and adhesive energy between RCA and asphalt. A small CA and great adhesive energy indicated good water stability.

The variation law of the fatigue performance of HMA-RCAs along with RCA content was determined by the interaction of the adhesion and porosity effects. Although the CA and adhesion energy between RAC and asphalt was similar to that of serpentine, the porosity of asphalt concrete increases with the increase of RCA. Therefore, when the aggregate contains more RCA, the fatigue performance of the HMA-RCA will decrease.

## Figures and Tables

**Table 1:** Technique performance of asphalts.

Asphalts Type	Penetration/0.1 mm	Ductility/cm	Brookfield Viscosity/(Pa·s)	Softening Point/°C	Density at 15 °C/(g/cm^3^)
Matrix asphalt	66.0	>150	0.486	47.5	1.018
SBS modified	72.3	42.3	1.345	71.0	1.033

Note: The testing temperatures of the ductility of the unmodified and SBS modified bituminous binder were 15 °C and 5 °C, respectively. The Brookfield viscosity at 135 °C was tested for the 70# matrix asphalt and SBS modified asphalt.

**Table 2:** Technique performance of aggregates.

Aggregate Type	Crushed Value/%	Apparent Relative Density				Water Absorption/%		
		0–5 mm	5–10 mm	10–20 mm	20–30 mm	5–10 mm	10–20 mm	20–30 mm
RCA	26.7	2.483	2.657	2.684	2.838	6.2	5.3	4.8
Activated RCA	21.8	2.523	2.707	2.731	2.845	1.9	1.8	1.1
Limestone	20.9	2.652	2.698	2.705	2.713	1.7	0.9	0.1
Granite	13.4	2.602	2.668	2.675	2.660	1.3	1.2	0.7
Serpentine	4.8	2.639	2.728	2.690	2.714	0.9	0.4	0.5

Note: The crush value refers to the performance index of the aggregate against crushing. It is used to measure the ability of the stone to resist crushing under the increasing load. The ratio of the weight of the crushed aggregate to the total weight of the sample is measured by a prescribed test method, expressed as a percentage.

**Table 3:** Parameters of the surface energy of water and ethyl alcohol.

Liquid Types	Surface ENERGy γ_l_/(10^−3^ J/m^2^)	Dispersion Component of Surface Energy *γ*_l_^d^/(10^−3^ J/m^2^)	Polar Component of Surface Energy *γ*_l_^p^/(10^−3^ J/m^2^)
Water	72.8	21.8	51
Ethyl alcohol	48.3	29.3	19

**Table 4:** Marshall indices of mixtures.

Mixture	Asphalt Content/%	Bulk Relative Density	VV/%	VMA/%	VFA/%	Effective Asphalt Content/%	Oil Absorbing Content/%	Stability/KN	Flow Value/0.1 mm
M1	3.57	2.462	4.4	12.21	63.97	3.45	0.12	11.2	23.5
M2	4.12	2.409	4.3	12.14	64.53	3.53	0.59	10.5	31.9
M3	5.12	2.335	4.3	12.99	66.76	4.04	1.08	9.8	30.1
M4	6.98	2.315	4.8	12.03	59.80	3.38	3.60	8.5	37.8
Limit value			3–6	≥12.0 (when VV = 4.0%)	55–70	-	-	≥7.5	15–40

Note: VV. air voids content; VMA: voids in mineral aggregate; VFA: voids filled with asphalt. According to the Chinese Specification of JTG F40-2004 (RIOH, 2004.) [33], the oil absorbing content can be calculated according to the density parameters of the aggregate, asphalt, and asphalt mixture.

**Table 5:** Aggregate types and numbers of different asphalt mixtures.

Aggregate Type	Granite			Serpentinite			Limestone			RCA
RCA content/%	60	30	0	60	30	0	60	30	0	100
Number	F1	F2	F3	F4	F5	F6	F7	F8	F9	F10

**Table 6:** CA results of Aggregates and solid asphalts.

Types of Aggregates and Solid Asphalts	Testing Liquid	
	Water	Ethyl Alcohol
Limestone	39.719	43.002
Granite	16.791	35.903
Serpentine	30.167	40.076
RCA	27.273	39.543
Matrix asphalt	93.649	74.269
SBS modified asphalt	84.163	71.924

**Table 7:** Surface energy of aggregates and solid asphalts.

Types of Aggregates and Solid Asphalts	*γ_s_^d^*/(10^−3^ J/m^2^)	*γ_s_^p^*/(10^−3^ J/m^2^)	*γ_s_ = γ_s_^p^ + γ_s_^d^*/(10^−3^ J/m^2^)
Limestone	0.955	70.202	71.16
Granite	0.008	98.397	98.40
Serpentine	0.221	84.579	84.80
RCA	0.099	88.760	88.86
Matrix asphalt	14.901	5.057	19.96
SBS modified asphalt	7.817	14.344	22.16

**Table 8:** CA between liquid asphalt and aggregate.

Aggregates	Matrix Asphalt	SBS Modified Asphalt
Limestone	25.182	28.925
Granite	32.515	40.810
Serpentine	26.916	30.094
RCA	29.192	32.948

**Table 9:** Adhesion energy between asphalt and aggregate.

Aggregates	Matrix Asphalt	SBS Modified Asphalt
Limestone	38.020	41.559
Granite	36.789	38.935
Serpentine	37.755	41.336
RCA	37.382	40.759

**Table 10:** Results of the fatigue life.

Stress Level	Fatigue Life/(cycle)									
	F1	F2	F3	F4	F5	F6	F7	F8	F9	F10
0.8	6124	8012	13254	5698	10320	12124	6917	9987	12798	3587
0.75	8945	9648	13715	6796	11230	14561	7523	11245	15243	4532
0.7	12345	17269	20147	11026	17865	21453	16248	17742	22178	5901
